# Facilitating the development of urgently required combination vaccines

**DOI:** 10.1016/S2214-109X(24)00092-5

**Published:** 2024-04-15

**Authors:** William P Hausdorff, Shabir A Madhi, Gagandeep Kang, Lassané Kaboré, Marta Tufet Bayona, Birgitte K Giersing

**Affiliations:** a Center for Vaccine Innovation and Access, PATH, Washington, DC, USA; b Faculty of Medicine, Université Libre de Bruxelles, Brussels, Belgium; c South African Medical Research Council Vaccines and Infectious Diseases Analytics Research Unit, University of the Witwatersrand, Johannesburg, South Africa; d Bill & Melinda Gates Foundation, Seattle, WA, USA; e PATH, Center for Vaccine Innovation and Access, Dakar, Senegal; f Gavi, The Vaccine Alliance, Geneva, Switzerland; g WHO Department of Immunization, Vaccines and Biologicals, Geneva, Switzerland

## Abstract

The essence of a vaccine lies in its ability to elicit a set of immune responses specifically directed at a particular pathogen. Accordingly, vaccines were historically designed, developed, registered, recommended, procured, and administered as monopathogen formulations. Nonetheless, the control and elimination of an astonishing number of diseases was realised only after several once-separate vaccines were provided as combinations. Unfortunately, the current superabundance of recommended and pipeline vaccines is now at odds with the number of acceptable vaccine administrations and feasible health-care visits for vaccine recipients and health-care providers. Yet, few new combinations are in development because, in addition to the scientific and manufacturing hurdles intrinsic to coformulation, developers face a gauntlet of regulatory, policy, and commercialisation obstacles in a milieu still largely designed for monopathogen vaccines. We argue here that national policy makers and public health agencies should prospectively identify and advocate for the development of new multipathogen combination vaccines, and suggest ways to accelerate the regulatory pathways to licensure of combinations and other concrete, innovative steps to mitigate current obstacles.

## The success and saturation of immunisation programmes

The range and breadth of human diseases that vaccines routinely protect against are not widely recognised. Vaccines can prevent more than 27 life-threatening conditions and an estimated 4–5 million deaths each year across the globe.^1^ Vaccines for other infectious diseases are urgently needed, and many might be available by 2030 (appendix p 2).^2^ But immunisation programmes that deliver these vaccines are becoming saturated. Without a fundamental rethinking of how vaccines are recommended, developed, and delivered, it is not clear how many more diseases we will be able to prevent—or even if we can maintain current disease prevention levels.

Vaccines introduced worldwide almost 40 years ago have brought polio to the brink of eradication and achieved measles elimination—at least temporarily—in some countries and regions. Vaccines have markedly decreased diphtheria, pertussis, and tetanus cases, and prevent disseminated and pulmonary tuberculosis in young children.

Today, vaccines protect against a lethal cause of dehydrating diarrhoea in infants (rotavirus) as well as rubella, a major source of congenital birth defects. Vaccines also prevent what were, before vaccination, major causes of debilitating and often deadly sepsis, bacterial meningitis, and pneumonia in young children (*Haemophilus influenzae* type b [Hib] and *Streptococcus pneumoniae*), as well as essentially eliminating the devastating epidemics of meningococcal meningitis caused by *Neisseria meningitidis* serogroup A, which routinely afflicted entire populations across a large swathe of sub-Saharan Africa. In high-income countries, vaccines targeting additional meningococcal serogroups B, C, W, and Y are dramatically reducing the (much lower) number of cases of this particularly fulminant form of meningitis that affects children, adolescents, and adults.

Hepatitis B and human papillomavirus (HPV) vaccines given to children and adolescents are preventing a substantial portion of liver and cervical cancers, respectively, in adults. New vaccines are being rolled out against malaria, still a major killer of children in Africa, and other diseases of regional importance such as dengue fever and typhoid fever. Alongside non-pharmacological interventions, vaccines against influenza and COVID-19 have been crucial for rapid and effective responses to pandemic threats. The collective effect of vaccination in preventing illness and death is enormous,^3^ and it does so in an extraordinarily cost-effective—and sometimes cost-saving—manner.^4^

These highly successful infant immunisation programmes have nonetheless had a limited effect on diseases that occur in infants younger than 3 months (with the notable exception of pneumococcal vaccination).^5^ Consequently, half of all child deaths occur during the neonatal period,^1^ when children's immune systems are too immature to fight infection or respond quickly and robustly to most vaccines. However, the first vaccine for maternal immunisation to target respiratory disease caused by respiratory syncytial virus in neonates and infants has been licensed,^6^ and vaccines to prevent pneumonia, sepsis, and meningitis caused by group B *Streptococcus* might not be far behind.^7^ Each is intended to be administered to pregnant people alongside other vaccines such as those for influenza, pertussis, tetanus, and COVID-19 currently protecting them and their newborns.

Furthermore, as COVID-19 (and Ebola virus disease, on a much smaller scale, before it) illustrated, there remains great public demand for vaccines to combat unpredictable and emerging diseases. Another role increasingly recognised for vaccines is in the control of antimicrobial resistance. For example, well known conditions such as typhoid fever, dysentery, various hospital-acquired and bacterial skin infections, gonorrhoea, and tuberculosis are becoming increasingly untreatable by antimicrobial agents but could be prevented by vaccines under development and broader deployment of existing ones. Immunisation is equally effective against susceptible and resistant organisms because the antigens that vaccines target are not those that cause antimicrobial resistance.^8^

Fortunately, the technical feasibility of developing new vaccines is high due to vaccine developers' increasing success in adapting and improving on older approaches (eg, polysaccharide-protein conjugation, outer membrane vesicles, and immunity-enhancing adjuvants), as well as the coming of age of novel vaccine platforms such as viral vectors and mRNA.

### The reality of current immunisation programmes

Unfortunately, immunisation programmes across the globe are struggling with the practicalities of accommodating the ever-increasing number of vaccines, the ancillary supplies needed to administer them within the confines of a limited number of health-care visits, and restricted budgets (table).

**Table:** The evolution in number of vaccines recommended both globally and regionally at different visits

	**1984**	**2010**	**2023**	**2030**
Maternal	..	1	1 +1 or more regional vaccines	2 +1 or more regional vaccines
Birth	1	2	2	2
Infant (aged 6 weeks to 6 months; 2 or 3 visits within 14 weeks)	2	3–5	3–6* +1 or more regional vaccines	3–6* +1 or more regional vaccines
6 months	..	..	+1 or more regional vaccines	+1 or more regional vaccines
7 months	..	..	+1 or more regional vaccines	+1 or more regional vaccines
9 months	1	2 +1 or more regional vaccines	2 +1 or more regional vaccines	2 +1 or more regional vaccines
15 months	..	1	1 +1 or more regional vaccines	1 +1 or more regional vaccines
12–23 months	..	2–4	1–4 +1 or more regional vaccines	1–4 +1 or more regional vaccines
Childhood (24 months to 8 years)	..	2–4	1–4 +1 or more regional vaccines	1–4 +1 or more regional vaccines
Adolescents (aged ≥9 years)	..	3–5	2–5 +1 or more regional vaccines	2–5 +1 or more regional vaccines
Adults	..	+1 or more regional vaccines	+1 or more regional vaccines	+1 or more regional vaccines
Older adults (aged ≥65 years)	..	..	+1 or more regional vaccines	+1 or more regional vaccines

This table reflects the number of separate administrations, per visit, of vaccines recommended globally by WHO;^9^ we also note at which visits one or more additional vaccines are recommended for high-burden regions or populations at high risk (termed regional vaccines). The specific vaccines underlying these numbers are shown in the appendix (p 2). Not all vaccines are administered parenterally. For clarity, the 2030 column only depicts the additional vaccines that we anticipate to be recommended in addition to those shown in 2023.

* In some cases, up to five or six vaccines are recommended but generally no more than four parenteral vaccines would be administered in a single visit.

At least six vaccination visits are required in the first 2 years of life to receive the ten routine vaccinations currently globally recommended by WHO in that age period, in addition to whatever vaccines are recommended for specific regions.^9^ Although the advent of a hexavalent combination vaccine in 2023 containing the inactivated polio vaccine has offered the prospect of decreasing the number of separate administrations required at some visits by children and adults, multiple vaccines are now offered at almost every visit (table; appendix p 3).

Nigeria, for example, offers four vaccines at each of the 6, 10, and 14-week visits; three separate parenteral vaccines are given at 14 weeks and at 9 months.^10^ US providers routinely offer three, four, or even five separate vaccines at a single visit, of which four can be parenterally administered.^11^ Most countries have similar regimens, with many adding region-specific vaccines targeting Japanese encephalitis, meningococcal meningitis, and yellow fever. The logistical complexity of delivering so many vaccines is well known to countries, with an especially large number of region-specific vaccines offered at age 9 months (appendix p 3).

Some patients and caregivers, when confronted with the expectation for multiple injections at a single visit, could decline or opt to come back later.^12^ Health-care provider attitudes towards multiple injections, if they overestimate parental concerns,^13^ can also affect uptake of recommended vaccines. But a missed or a delayed immunisation leaves infants susceptible to disease, and in too many cases the deferred visit will not occur at all because of practical difficulties and costs incurred in returning to the health centre.^14^, ^15^ As more injections are proposed to vaccine-hesitant adolescents, adults, and people who are pregnant, strategies to enhance vaccine confidence will need to form an intrinsic part of new introduction and implementation strategies.

Furthermore, the increasing numbers of vaccines individually administered can also be financially prohibitive for many governments, with costs to fully vaccinate a child through the second year of life ranging anywhere from US$37 to $101 in low-income and middle-income countries (LMICs), depending on which vaccines the country decides to introduce, the price negotiated with the supplier, and the cost to deliver and administer the vaccine within their health-care system.^16^ A substantial portion of immunisation programme costs, especially in LMICs, is associated with refrigerated storage and transport, training, demand generation efforts, injection supplies, and service delivery.^17^ For+example, these costs account for a third to half of the incremental costs of introducing a new, standalone typhoid conjugate vaccine in Malawi.^18^ The incremental supply chain and service delivery costs in particular would be reduced, if not eliminated, if the typhoid conjugate vaccine was offered in combination with another vaccine already being provided, in part because such a combination would occupy much less refrigerated storage space and require half the syringes as the two standalone vaccines. For this reason, up to nine vaccines are now available as two combinations (diphtheria, tetanus, pertussis, polio, Hib, and hepatitis B; measles and rubella [with or without mumps]), reducing both immunisation programme costs and the number of injections an infant needs to receive in a single visit.^19^

Most of the vaccines recommended for use in the last decade have been targeted to specific high-burden regions or populations at high risk, such as dengue fever vaccine and typhoid conjugate vaccine. Similarly, with a few prominent exceptions (pandemic [eg, influenza and COVID-19], HIV, and new tuberculosis and malaria vaccines), most new vaccines in development are targeted at pathogens that individually cause moderate levels of mortality relative to pathogens targeted by the globally recommended vaccines, such as HPV. New so-called moderate-burden vaccines might not be globally recommended, but rather deployed in specific subregional or subnational contexts. The potentially small market size of moderate-burden new vaccines casts doubt on their eventual uptake and commercial viability as separate injections. Several vaccines prioritised by public health experts,^20^ including some with a high probability of technical success, such as a *Shigella* vaccine,^21^ have thus not attracted public commitments by commercial manufacturers to take them through full development.

## Multipathogen combination vaccine development: a solution

Multipathogen combination vaccines—that is, more than one vaccine in the same injection to tackle multiple diseases—are compelling because they could substantially increase adherence to vaccination schedules through easier delivery and greater acceptability from caregivers, health-care providers, and vaccine recipients. Combination vaccines are also naturally attractive to those conducting immunisation programmes because they are easier, quicker, and cheaper to administer, with few errors and decreased syringe and vial disposal requirements.^19^ However, they also present some challenges (panel 1).

Panel 1 **Potential value of a combination vaccine versus coadministration of separate antigens**

**Vaccine delivery**

*Potential advantages*

• Improved timeliness of vaccination; greater acceptability by end users and health-care providers
• Higher and more equitable vaccination coverage

*Potential challenges*

• Need to ensure compatibility of vaccination schedules and routes of administration of each component

**Health impact**

*Potential advantages*

• Greater, more equitable impact
• Facilitates targeting less prevalent but still important pathogens
• Allows possibility of a syndromic combination—ie, a combination vaccine targeting pathogens causing same clinical syndrome

*Potential challenges*

• Need to articulate and show incremental health and economic value of overall combination compared with stand-alone components
• More difficult to attribute a safety signal to any particular component

**Vaccine administration efficiency and cost**

*Potential advantages*

• Fewer syringes
• Fewer packaging disposal needs
• Less cold chain storage and transportation space required
• Shorter administration time and errors
• Fewer needlestick injuries and therefore improved safety for vaccinators

*Potential challenges*

• Could be more expensive to procure than total of component vaccines, even if less expensive to deliver
• Available combinations could contain more vaccines than countries prefer to introduce

**Vaccine design, development, and supply**

*Potential advantages*

• Potentially greater demand for combination than individual components, leading to economies of scale and reduced cost of goods

*Potential challenges*

• Currently little guidance or recommendations from the public health community regarding combination composition or use preference
• Greater risk of failure due to immunological interference or unacceptable reactogenicity
• More complex, lengthy, and expensive clinical development pathway due to current regulatory guidelines
• Need to develop and validate additional assays to accurately characterise components within complex mixtures
• Could restrict competition if few developers have access to all necessary components or need to enter into complex intellectual property rights agreements
• In the absence of guidance on preferred combination components, could be difficult to avoid a plethora of competing, overlapping, and commercially unviable combinations due to market fragmentation, ultimately increasing cost

The theoretical attraction of combination formulations was apparent in a 2022 mixed-methods study of vaccine preferences. In this study, 70 experts in six different LMICs were asked to choose between two hypothetical rotavirus vaccine formulations for young children.^22^ Regardless of country, 80% strongly preferred a moderate efficacy vaccine combined with their current formulation containing diphtheria, tetanus, pertussis, Hib, and hepatitis B, over an oral formulation offering much higher clinical protection but requiring three doses delivered at separate visits.^22^ The same investigators reported that community-facing health-care providers are enthusiastic about being able to provide greater rotavirus protection, but not with more injections.^23^

There are few alternatives to combination vaccines. In LMICs, new vaccines could take advantage of the increasing number of contacts with health-care providers created to allow introduction of multidose malaria vaccination in infants, boosters of measles, diphtheria, tetanus, and pertussis in the second year of life, and adolescent visits for HPV vaccines. Alternative delivery technologies, such as needleless injectors, microarray patches, and controlled-release formulations (that would effectively convert, for example, a three-dose vaccine to a single-dose vaccine) could also help, but most of these innovations are in early development and have a complex policy, regulatory, and introduction pathway to navigate.^24^

Coformulation of separate vaccines into a single combination can have an almost existential effect, transforming some vaccines with very low uptake (and thus minimal public health impact) into powerful interventions responsible for major decreases in important diseases globally. For example, the true potential effect of vaccination against Hib pneumonia and meningitis was only realised once it was co-formulated with existing vaccines for diphtheria, tetanus, pertussis, and hepatitis B, and a comparable effect was observed for rubella once combined with the measles vaccine. The ability to provide broader disease protection without additional injections led to many more countries choosing to adopt Hib and rubella in their immunisation programmes.^25^ Similarly, it seems questionable whether countries will ever broadly introduce a standalone vaccine, if developed, against the rare but devastating congenital Zika syndrome, unless it has the option of being delivered in combination with a vaccine that is clearly in demand, such as one targeting dengue fever.

Combination vaccines designed to provide broad protection against multiple pathogens underlying a single clinical syndrome, or whose cumulative effects result in common long-term sequelae, could have important benefits. For example, a combination vaccine targeting the multiple enteric diarrhoeal pathogens strongly associated with linear growth faltering and stunting could produce much more profound effects on these outcomes than might be detected with vaccines targeting one or two major pathogens. Similarly, a vaccine against the major bacterial causes of otitis media could lead to synergistic reductions in antimicrobial use, as health-care professionals might choose to delay offering antibiotics to a patient with a middle ear infection knowing the patient had previously been vaccinated against the most serious causes of this condition. The overall value of a syndromic combination could also be easier to communicate to the public than the individual value of each of the component vaccines (appendix p 4)*.*

As an alternative or addition to the syndromic approach, vaccine combinations that are centred around existing immunisation programme visits (as previous combinations have been) could facilitate equitable coverage to target populations, such as adolescents and adults, who are currently difficult to reach in many countries.

## Inherent risks in combination vaccine development

Despite these benefits, there is currently a low commercial appetite to develop vaccine combinations targeting multiple pathogens. Only a few are licensed, and a few others are currently in advanced stages of clinical development.^26^, ^27^, ^28^ The paucity of combination vaccine candidates reflects the fact that developers perceive that the technical and commercial risks and costs of combination vaccine development outweigh the potential return on investment.

The barriers to the development, licensure, and commercial viability of combinations are myriad. First, there is an absence of guidance by any internationally recognised group of experts that identifies the components of desired future combination vaccines. Vaccine developers are thus left to guess the identity of these components, and incorrect guesses are costly and discouraging.^29^ Second, combination vaccine developers need access to multiple components, which could pose substantial commercial and intellectual property complexities. Even when vaccine developers have the components in hand, developers must overcome sometimes daunting scientific and manufacturing challenges to physically combine vaccines—including designing or adapting complex analytical tests to see whether the individual components interact with, modify, or affect each other. The development of mRNA and viral vector platforms could facilitate inclusion of nucleic acids encoding vaccine antigens and thereby target multiple pathogens within a single construct or backbone;^30^ however, developers will still need to ensure, as with all combination vaccines, that after immunisation there is no clinically relevant immunological interference between antigens. Third, the clinical development and regulatory pathways currently used to ensure vaccine efficacy, quality, and safety were designed for single pathogen vaccines; combinations are viewed as essentially cumulative versions of individual vaccines, where every component, no matter how minor, should meet the same rigorous efficacy criteria to justify its inclusion. This perspective makes licensure of multiantigen combination vaccines quite challenging, compared with individual vaccines.

In general, when a vaccine is targeted at a less frequently circulating, but nonetheless deadly pathogen, it is not feasible to conduct clinical disease-preventing trials because there are not enough cases to measure the difference between the vaccine and placebo groups. Licensure is instead usually based on the safety and ability of the candidate vaccine to stimulate immune responses similar to those elicited by an existing vaccine—assuming one exists. Yet in the case of multi-component combination vaccines these comparisons become particularly complex. The increased number of immunological comparisons due to the multiplicity of components inherently increase the likelihood that one or more component, even if quite immunogenic, will statistically fail a non-inferiority comparison, even by chance—especially if each component itself comprises multiple antigens. Unless a quantitative threshold correlating a given immune response with clinical protection has been established for each component, it is unclear what the clinical relevance of a decreased response might be. Uncertainty as to how regulators might interpret decreased immunological responses in one, perhaps relatively minor, component increases the risk that a developer will abandon the entire, multipathogen product, even if the overall clinical effect of the combination is anticipated to be quite positive.

A key prerequisite for broad uptake and commercial success of any vaccine is whether it has secured recommendations for use from WHO, based on advice from its Strategic Advisory Group of Experts on Immunisation (SAGE). However, even for combination vaccines that have been proven safe, effective, and are licensed, current global policy and recommending bodies do not preferentially advocate for combination vaccines, such as the combination of diphtheria, tetanus, pertussis, Hib, and hepatitis B,^30^, ^31^ or measles and rubella,^32^, ^33^ over standalone vaccines.^25^ Rather, WHO recommendations currently call for vaccination against each of the individual diseases without suggesting any preference for combination formulations.

The absence of a preference for combinations matters because global policy recommendations from WHO inform policy recommendations at the country level by national immunisation technical advisory groups (NITAGs). WHO policy recommendations are also crucial for Gavi, the Vaccine Alliance, who finances and subsidises the procurement of vaccines for half the world's children. But of course, countries themselves ultimately decide which of the WHO-recommended or Gavi-eligible vaccines they will introduce, based on their own evaluation of their priorities, budget, and epidemiological context. NITAGs in countries not reliant on WHO SAGE recommendations, such as the US Advisory Committee on Immunization Practices, can also be hesitant to express preferences for combination vaccines over administration of their individual components. This hesitancy was evident in recent deliberations regarding a newly licensed vaccine offering protection against all five meningococcal serogroups, even though a preferential recommendation might help mitigate the currently sub-optimal immunisation coverage due to administration of two separate vaccines.^34^

Unintentionally, the current absence of a clearly articulated preference by policy makers for combination vaccines is interpreted by vaccine developers to mean that neither the major effort and investment required to develop a combination formulation, nor the incremental public health and economic value of such a formulation in enhancing the breadth and extent of vaccine programmes, are inherently valued. A focus on the merits of individual vaccines, without considering that how they are formulated could affect the overall acceptance of vaccines and functioning of immunisation programmes, is ironic considering the widely accepted principle that vaccines do not prevent disease, only vaccination does.

## Monopathogen combination vaccines as role models

Some of the authors of this Health Policy helped devise the policy and regulatory pathways that successfully catalysed the development, licensure, and availability of pneumococcal conjugate vaccines—highly complex monopathogen combination formulations in their own right. We believe that these pathways could lead the way to an alternative approach to the development of multipathogen combination vaccines. The licensure pathway followed for another monopathogen combination, multivalent meningococcal conjugate vaccines, also offers useful lessons.

In each case, the vaccine developers were seeking to license multivalent protein-polysaccharide conjugate formulations, which require eliciting specific immune responses against each one of the most prominent disease-causing strains. Unfortunately, each of the more than 100 pneumococcal serotypes identified can, as far as we know, cause disease. Therefore, having different developers choose a manageable subset of the most important serotypes to target in their vaccine formulation could result in a confusing array of overlapping formulations. In addition, although coformulation of multiple serotype-specific conjugates might appear straightforward, the considerable immunological interference observed between conjugates using the same carrier proteins (as found in coadministered combination diphtheria, tetanus, and acellular pertussis formulation) has condemned some early attempts to develop pneumococcal conjugate vaccines.^35^ To provide guidance to vaccine developers, international experts under the auspices of WHO and Gavi defined a minimally acceptable pneumococcal conjugate vaccine composition,^36^ based on current pneumococcal serotype epidemiology. These guidelines, along with a sizeable amount of funding (also known as an advance market commitment) set aside for pneumococcal conjugate vaccine procurement, gave both a design target and evidence of a tangible commercial demand, and therefore a clear incentive, to developers.

In the case of meningococcal disease, five of the six disease-causing strains (serogroups A, C, W, Y, and X) are potential targets for a multivalent conjugate formulation (for immunological reasons, vaccines against meningococcal serogroup B required a more complex, non-conjugate vaccine development approach).^37^ Because each of the six strains show important temporal and geographical variation, such as a virtual absence of serogroup A disease in much of the world despite causing epidemics in Africa,^38^ in the absence of global agreement on meningococcal vaccine serogroup composition, meningococcal conjugate vaccines containing one, two, four, and—in 2023—all five serogroups^39^ were ultimately developed. However, for both pneumococcal conjugate vaccines and meningococcal conjugates, how to show that each component was in itself clinically effective posed the next major challenge, as many strains are too rare for studies to show clinical protection.

A pivotal clinical study involving 40 000 children who had received a heptavalent pneumococcal conjugate vaccine candidate was designed to evaluate protection against invasive pneumococcal disease caused by any of the seven vaccine serotypes. Although the vaccine candidate ultimately proved to be 100% effective, clinical efficacy in this study could only be shown against disease caused by the three most common serotypes. Nonetheless, the licence granted by regulators explicitly covered all seven, presumably because each serotype component elicited robust immune responses against the capsular polysaccharide and was deemed likely to confer clinical protection.^40^ Granting the licence meant acceptance of an amount of risk, as the precise level of immune response needed was unknown—ie, there is no established protective immunological threshold against disease caused by any serotype. Subsequent postlicensure impact data with the heptavalent vaccine along with efficacy data, effectiveness data, or both from nonavalent and decavalent formulations with similar immunogenicity levels validated this decision, as they showed very high levels of clinical protection, with no evidence of low efficacy for any serotype.^41^, ^42^

For the highly desirable subsequent generations of pneumococcal conjugate vaccines containing even more (>7) components, a precise immune threshold was needed to allow serotype-specific comparisons of the vaccine candidate with a licensed product; however, no such threshold existed for any pneumococcal serotype. For simplicity, the threshold chosen, after intensive expert consultation, represented a very gross approximation across all serotypes of what might be an average protective level^43^—even though the true clinically protective levels likely vary from serotype to serotype by as much as a factor of ten.^44^

Finally, almost every pneumococcal conjugate vaccine formulation has had one or more immunogenic serotype that did not show statistical non-inferiority compared with the licensed comparator. Because each pneumococcal conjugate vaccine, overall, was considered of major public health value, regulators still granted licensure. Although not every serotype has subsequently been shown to be more than 90% effective in postlicensure surveillance studies, the overall impact of pneumococcal conjugate vaccines on pneumococcal disease in children has been very impressive.^41^

For meningococcal conjugates, however, a clinical efficacy study was not possible due to the rarity of meningococcal disease, even in aggregate, and regulators had to rely on strain-specific immune responses (against the polysaccharide capsule) that they knew were bactericidal. Although experts agreed that this type of functional immune response against meningococcus would likely confer protection, the actual threshold needed was unknown except for serogroup C.^38^, ^45^ To move forward, the pragmatic assumption was made that the threshold for serogroup C would be used for each of the other serogroups in this vaccine.^38^, ^45^ This threshold was met, multivalent meningococcal conjugate vaccines were licensed, and subsequent postlicensure impact data have indicated high effectiveness against disease caused by all three new serogroups.^46^, ^47^, ^48^, ^49^, ^50^

In summary, to facilitate the availability of high priority but highly complex pneumococcal and meningococcal combination vaccines, several innovative steps were taken (panel 2). It is recognised that some of these steps were helped by general agreement that the nature of the protective immune response (eg, anti-capsular antibodies) was the same for all pneumococcal serotypes or all meningococcal serogroups, and that these were linked to functional activity (opsonophagocytic and bactericidal, respectively). This situation would differ for some desirable multipathogen combinations, for which even the nature of the protective immune response could be unknown. This challenge highlights the need for a more concerted focus on and support for determining mechanistic correlates of protection against diseases caused by pathogens envisioned to be included in combination vaccines.^52^ Nonetheless, we note that even with pneumococcal and meningococcal conjugates, the necessary antibody threshold to provide clinical protection remained undetermined, but that this did not pose an insurmountable barrier to their licensure. We suggest that this tolerance of uncertainty could serve as a foundation for facilitating development and availability of multipathogen vaccines.

Panel 2 **Innovative steps taken in the development of pneumococcal and meningococcal combination vaccines**

• Consensus was reached on the minimum core components (ie, serotypes) of multivalent (albeit monopathogen) pneumococcal combinations to be included in a formulation eligible for broad recommendations by WHO^51^ and for purchase via an advanced market commitment mechanism by Gavi
• Innovative regulatory guidelines were established that took into account the practical impossibility of showing a high level of clinical efficacy for each component of pneumococcal conjugate vaccines or meningococcal conjugate vaccines before licensure, and using (for pragmatic reasons) approximate thresholds of protective immunity as surrogates
• There was acceptance of the possibility (for pneumococcal conjugate vaccines) that multiple immunological comparisons could result in some immunogenic components not reaching statistical non-inferiority comparisons, yet still allow licensure of the product as a whole

## Conclusion

Novel and proactively designed development, regulatory, and policy approaches are needed if we are to successfully deliver many of the vaccines in the current pipeline, and even to help sustain current programmes. Specifically, we advocate for: extensive country and regional consultations to identify and validate the priority vaccine targets that could form the basis of a comprehensive combination strategy; assessment of the full public health and socioeconomic value of new vaccines, both as individual components as well as their incremental value as combination vaccines, to inform investment and introductory decision making; WHO to leverage its advisory committees, including SAGE, to evaluate the potential vaccine combination strategies and consider signalling the priority combinations based on regional preferences, public health need, and socioeconomic impact; international financing and procurement agencies such as Gavi and UNICEF should include novel combination vaccines within their market shaping strategies, based on country and regional preferences and supportive statements from WHO; regulatory authorities to give more weight to the overall clinical benefit of combination vaccines and to the practical advantages of minimising the number of vaccine administrations (ie, improved and more equitable coverage) in licensing decisions, without compromising on safety; insurance companies that allow a choice between use of a combination and the separate component vaccines to ensure that per-vaccine reimbursement policies do not inadvertently incentivise the health-care provider to administer the standalone formulations; and early engagement with immunisation programme managers, providers, caregivers, and vaccine recipients to better assess the overall programmatic implications of combination vaccine administration (eg, potential reduced burden on systems and health providers), and to understand and address the perceived acceptability of combination vaccines versus co-administration of standalone components (panel 3). We need a framework shift in regulatory, policy, and financing perspectives that recognises the inherent value of combination vaccines, and moves away from considering them as essentially an equivalent to the sum of their individual parts. This shift will not be easy, but there might be little choice if we want to continue to rely on vaccine-based approaches to address some of the most noteworthy health challenges of our time.

Panel 3 **Tangible and innovative steps to accelerate availability of multipathogen combination vaccines**

• Extensive country and regional consultations to identify and validate the priority vaccine targets that could form the basis of a comprehensive combination strategy.
  • The potential combinations discussed will have to be limited to those that appear technically feasible and epidemiologically appropriate and could differ by region. These consultations might also weigh the relative merits of potential syndrome-specific vaccine combinations against combinations comprised of unrelated vaccines given at the same visit. Although these will be challenging exercises, engagement of disease experts and policy makers that constitute country and regional technical advisory groups could help to identify the most desirable configurations. Without this public health guidance, the few combinations that are developed will be based on the perceptions of vaccine manufacturers alone as to what might be commercially viable.
• Assessment of the full public health and socioeconomic value of new vaccines, both as individual components as well as their incremental value as combination vaccines, to inform investment and introductory decision making.
  • Evaluation of vaccine value beyond showing individual health benefits, to include broader socioeconomic and indirect effects such as the effect on equitable immunisation coverage and timeliness, disease transmission, improvements in equity, and the effect on immunisation programme efficiency, should be undertaken to evaluate the trade-offs in developing and delivering individual versus combination vaccines.^53^ These assessments will inform estimates of potential demand and market opportunity.
• WHO to leverage its advisory committees, including their Strategic Advisory Group of Experts on Immunisation, to evaluate the potential combination strategies and consider signalling the priority combinations based on regional preferences, public health need, and socioeconomic impact.
  • So as not to stifle competition and developer innovation, and to facilitate development of regional configurations, it might be preferable to define the crucial core target pathogens that should be coformulated and to which others might be added. For combinations that contain globally recommended vaccines, such as a hexavalent combination of diphtheria, tetanus, pertussis, *Haemophilus influenzae* type b, hepatitis B, and inactivated polio vaccine, WHO could consider preferentially recommending the combination vaccine over individual components to improve uptake and achieve greater vaccination coverage.
• International financing and procurement agencies such as Gavi and UNICEF to include novel combination vaccines within their market shaping strategies, based on country and regional preferences and supportive statements from WHO.
  • For example, Gavi periodically conducts rigorous assessments of new vaccines, including combinations, which are near licensure and being considered for procurement as part of its Vaccine Investment Strategy; it might expand those assessments to include potentially desirable combinations identified by WHO. Their inclusion could further incentivise combination vaccine developers.
• Regulatory authorities to give more weight to the overall clinical benefit of combinations and to the practical advantages of minimising the number of vaccine administrations (ie, improved and more equitable coverage) in licensing decisions, without compromising on safety.
  • This approach could allow regulators to justify lowering the level of clinical and statistical certainty regarding clinical efficacy—ie, not every single component of a combination might need to be shown to be highly protective against its target pathogen if the overall vaccine value is high. Increased, concerted investment from funding agencies for the development of mechanistic correlates of vaccine-induced protection, and to better predict immunological interference between candidate combination vaccine components, will also help facilitate vaccine development and regulatory approvals. Combination vaccines must, of course, continue to be subject to the same high safety standards as individual vaccines. Such assessment will help to diminish spurious claims (such as the repeatedly debunked association of the measles, mumps, and rubella vaccine with autism) that there is something intrinsically unsafe about combination formulations. Increased reliance on postlicensing studies will be necessary to assess the real-world effects of combination vaccines on disease effectiveness and vaccine safety.
• Insurance companies that allow a choice between use of a combination and the separate component vaccines to ensure that per-vaccine reimbursement policies do not inadvertently incentivise the health-care provider to administer the standalone formulations.
• Early engagement with immunisation programme managers, providers, caregivers, and vaccine recipients to better assess the overall programmatic implications (eg, potential reduced burden on systems and health providers), and to understand and address the perceived acceptability of combination vaccines versus co-administration of standalone components.

## Declaration of interests

WPH reports receiving a travel grant from GSK. SAM reports receiving funds from the Bill & Melinda Gates Foundation, GSK, Pfizer, Minervax, Novavax, Merck, Providence, Gritstone, and Immunity Bio. All other authors declare no competing interests.
