# Correction to *Lancet Glob Health* 2024; published online April 15. https://doi.org/10.1016/S2214-109X(24)00092-5

**DOI:** 10.1016/S2214-109X(24)00184-0

**Published:** 2024-04-25

*Hausdorff WP, Madhi SA, Kang G, Kaboré L, Tufet Bayone M, Giersing BK. Facilitating the development of urgently required combination vaccines.* Lancet Glob Health *2024; published online April 15. https://doi.org/10.1016/S2214-109X(24)00092-5*—In this Health Policy, the first sentence of paragraph 2 on page 5 should read “... based on advice from its Strategic Advisory Group of Experts on Immunisation”. The third bullet point of Panel 3 should read “WHO to leverage its advisory committees, including their Strategic Advisory Group of Experts on Immunisation”. This correction has been made as of April 25, 2024.

