# Supplementary material for: Facilitating the development of urgently required combination vaccines
Source: Lancet Glob Health. 2024 Apr 15;12(6):e1059–67. doi: 10.1016/S2214-109X(24)00092-5 (PMC11099297; doi:10.1016/S2214-109X(24)00092-5)
Supplement: Supplementary appendix [file mmc1.pdf]

THE LANCET
Global Health

Supplementary appendix

This appendix formed part of the original submission and has been peer reviewed. We post it as supplied by the authors.

Supplement to: Hausdorff WP, Madhi SA, Kang G, Kaboré L, Tufet Bayona M, Giersing BK. Facilitating the development of urgently required combination vaccines. *Lancet Glob Health* 2024; published online April 15. https://doi.org/10.1016/S2214-109X(24)00092-5.

APPENDIX TO:

Facilitating the development of urgently required combination vaccines by William P. Hausdorff, Shabir A. Madhi, Gagandeep Kang, Lassane Kabore, Marta Tufet Bayona, Birgitte K. Giersing

Figure 1. The increasing number of pathogens and diseases preventable by vaccination

	Maternal	Birth	Infants and toddlers	Childhood and adolescents	Adults and older adults
**1984:** 6 global diseases		• **Tuberculosis**	• **Pertussis**<br>• **Tetanus**<br>• **Polio**<br>• **Measles**<br>• **Diphtheria**		
**2010:** 11 global +5 regional diseases	• **Tetanus**	• **Hepatitis B**<br>• **Tuberculosis**	• **Diphtheria**<br>• **Tetanus**<br>• **Pertussis**<br>• **Hepatitis B**<br>• **Polio**<br>• **Measles**<br>• **Rubella**<br>• ***H. influenzae* b**<br>• **Rotavirus**<br>• **Pneumococcus**<br>• Meningococcus<br>• Mumps	• **Diphtheria**<br>• **Tetanus**<br>• **Pertussis**<br>• **Hepatitis B**<br>• **Polio**<br>• ***H. influenzae* b**<br>• **HPV**<br>• Meningococcus	• Zoster (Shingles)<br>• Yellow Fever<br>• Varicella<br>• Influenza<br>• Meningococcus
**2023:** 12 global +16 regional diseases	• **Tetanus**<br>• COVID-19<br>• Pertussis<br>• Influenza<br>• RSV	• **Hepatitis B**<br>• **TB**	• **Diphtheria**<br>• **Tetanus**<br>• **Pertussis**<br>• **Hepatitis B**<br>• **Polio**<br>• **Measles**<br>• **Rubella**<br>• ***H. influenzae* b**<br>• **Rotavirus**<br>• **Pneumococcus**<br>• Mumps<br>• Cholera<br>• Tick Borne Enceph<br>• Varicella<br>• Hepatitis A<br>• Jap. Encephalitis<br>• Typhoid<br>• Meningococcus<br>• Yellow Fever<br>• Malaria	• **Diphtheria**<br>• **Tetanus**<br>• **Pertussis**<br>• **Hepatitis B**<br>• ***H. influenzae* b**<br>• **HPV**<br>• COVID-19<br>• Influenza<br>• Meningococcus	• **Pneumococcal**<br>• Zoster (Shingles)<br>• RSV<br>• Dengue<br>• COVID-19<br>• Influenza<br>• Meningococcus
**2030:** Up to 30 diseases	• **GBS**<br>• **Tetanus**<br>• COVID-19<br>• Pertussis<br>• RSV<br>• Influenza<br>• CMV	• **Hepatitis B**<br>• **TB**	• **Diphtheria**<br>• **Tetanus**<br>• **Pertussis**<br>• **Hepatitis B**<br>• **Polio**<br>• **Measles**<br>• **Rubella**<br>• ***H. Influenzae* b**<br>• **Rotavirus**<br>• **Pneumococcus**<br>• RSV<br>• Mumps<br>• Cholera<br>• Tick Borne Enceph<br>• Varicella<br>• Hepatitis A<br>• Jap. Encephalitis<br>• Typhoid<br>• Meningococcus<br>• Yellow Fever<br>• Malaria<br>• Shigella	• **Diphtheria**<br>• **Tetanus**<br>• **Pertussis**<br>• **Hepatitis B**<br>• ***H. influenzae* b**<br>• **HPV**<br>• COVID-19<br>• Influenza<br>• Meningococcus<br>• Tuberculosis*	• **Pneumococcal**<br>• Zoster (Shingles)<br>• RSV<br>• Dengue<br>• COVID-19<br>• Influenza<br>• Meningococcus<br>• Tuberculosis*

Recommended schedule

Maternal · Infants and toddlers · Adults and older adults · Birth · Childhood and adolescents

Legend: The twelve pathogens and diseases in bold black font represent targets of globally recommended vaccines for routine vaccination, according to WHO[9]; those in plain font represent targets of vaccines recommended for high-burden contexts,regions and/ or high-risk populations. 2030 additions are presumed by the authors to be forthcoming but have not yet been formally recommended. Pathogens and diseases are grouped in differently colored squares that reflect the age ranges to whom each vaccine is administered. Abbreviations: Jap. Encephalitis: Japanese Encephalitis; RSV: Respiratory Syncytial Virus; HPV: Human Papilloma Virus; Tuberculosis*; Tuberculosis vaccine for adults and adolescents; Tick Borne Enceph.: Tick Borne Encephalitis; CMV: Cytomegalovirus.

Appendix Figure 2: **Evolution in vaccines recommended for use by WHO, by immunization visit.**

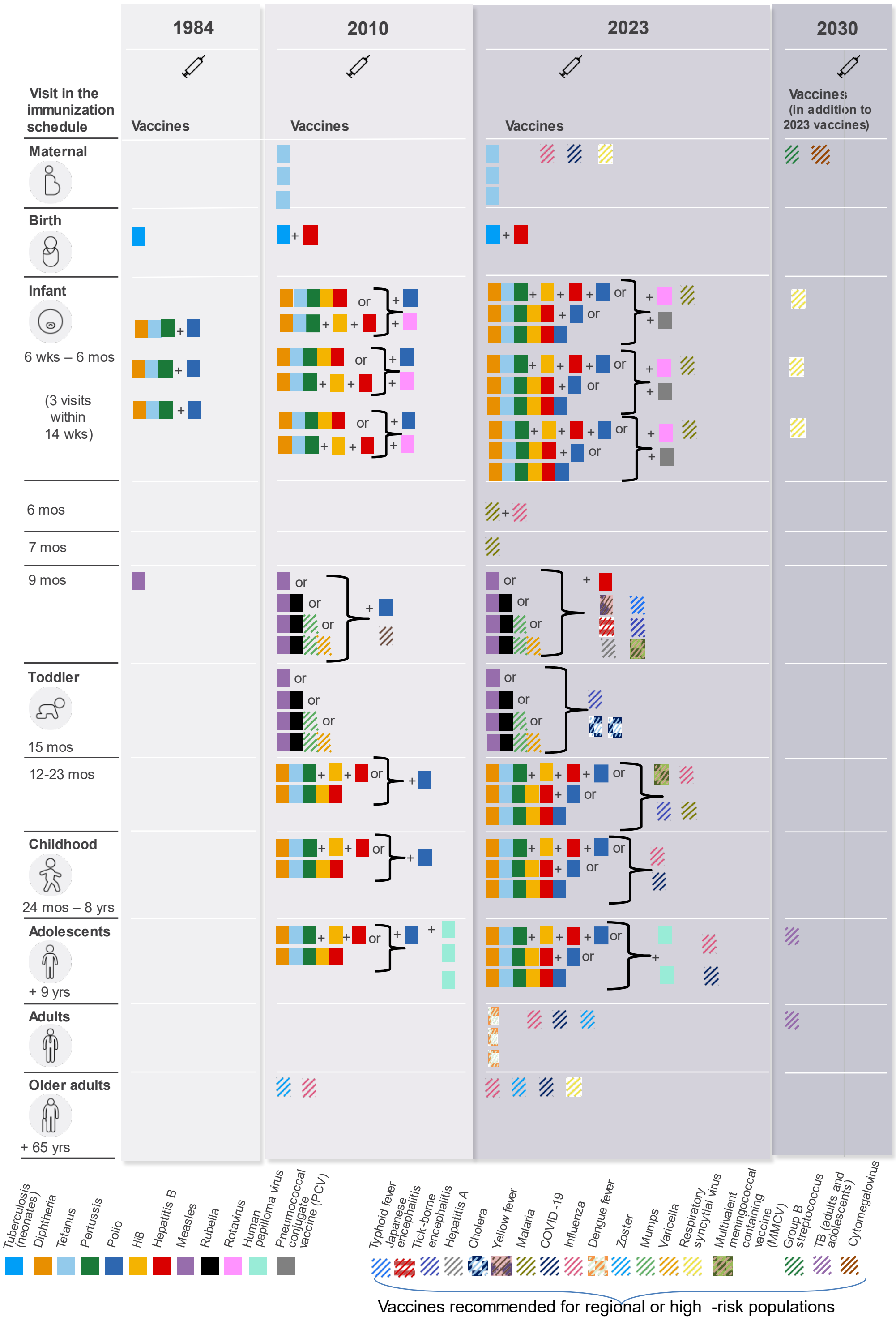

Legend: Boxes touching each other denote multi-pathogen combination vaccines. Solid boxes represent vaccines recommended by WHO globally, and hashed boxes represent vaccines recommended for high-risk and/or regional populations3. All vaccines are administered parenterally with the exception of rotavirus and certain polio, cholera, and influenza vaccines, For clarity, the 2030 column only depicts the vaccines hypothesized by the authors to be recommended *in addition* to those already recommended in 2023.

Table: Illustrative examples of syndromic combination vaccines

**Clinical Syndrome**	**Target Age Group**	**Possible Vaccine Component Targets**
Neonatal sepsis, meningitis, and respiratory disease	Pregnant people	Tetanus, Group B *Streptococcus*, RSV, *Klebsiella pneumoniae*, *Acinetobacter baumanii*, *B. pertussis*, influenza, COVID-19
Otitis Media (middle ear infections)	Infants	*S. pneumoniae*, non-typable *H. influenzae*, *M. catarrhalis*, RSV
Severe Diarrhea & Other Bacterial Enteric Infections	Infants and young children	*Shigella*, Enterotoxigenic *E. coli*, *Campylobacte*r, Typhoid, Paratyphoid, non-typhoidal salmonella, norovirus
Pneumonia	Infants, older adults	*S. pneumoniae*, non-typable *H. influenzae*, Metapneumovirus, COVID-19, Influenza, RSV, adenovirus
Emerging vector-borne diseases	All ages	Dengue, Zika, Chikungunya, Yellow fever, Japanese encephalitis

RSV = Respiratory Syncytial Virus
